# UVB, UVA, and visible light (blue-violet range) transmittance of clothing used in Brazil^⋆^^⋆⋆^

**DOI:** 10.1016/j.abd.2020.03.017

**Published:** 2020-09-12

**Authors:** Melissa Mari Yoshida, Ana Cláudia Cavalcante Esposito, Hélio Amante Miot

**Affiliations:** a Faculty of Medicina, Universidade Estadual Paulista, Botucatu, SP, Brazil; b Pathology Postgraduate Program, Universidade Estadual Paulista, Botucatu, SP, Brazil; c Department of Dermatology and Radiotherapy, Universidade Estadual Paulista, Botucatu, SP, Brazil

*Dear Editor,*

Photoprotection encompasses a series of behavioral measures that reduce exposure to solar radiation, such as knowledge of solar irradiation patterns (timetable, altitude, cloud coverage) and its reflection on surfaces, searching for shade, clothing, and the use of sunscreens.^1^

Most of the skin surface is usually covered by clothing and its different radiation transmittances can induce a false perception of safety, especially in children, individuals with lighter phototypes, those with photosensitive dermatoses, and populations at higher risk, such as workers exposed to the sun and immunocompromised individuals.^1^, ^2^ In the prevention and treatment of photoinduced or photoexacerbated dermatoses, it is important to understand the photoprotection provided by clothing.

The main biologically active solar radiations on the skin are ultraviolet B (UVB), ultraviolet A (UVA), and visible light (especially the blue-violet range: 400 − 500 nm), involved in inflammatory processes, dyschromias, photoaging, and carcinogenesis.^3^

Transmittance is defined as the percentage of radiation that passes through the matter, being complementary to the absorbance value. The knowledge of the transmittance of a fabric allows the calculation of its ultraviolet protection factor (UPF) for UVA and UVB.^4^ The transmittance of solar radiation through clothing depends on the density of the weave and thickness of the fabric. However, there are variations in this profile depending on the color, moisture of the clothing, type of fabric, and number of washes (since fabric wear increases transmittance). Some fibers receive specific treatments that impregnate photoprotective substances, increasing their UPF.^1^, ^5^

The transmittance of clothing used in Brazil to the main forms of solar radiation is unknown. This study aimed to evaluate the transmittance of UVB, UVA, and blue-violet light (400 − 500 nm range) in a series of garments. A total of 16 fabrics from commercially available clothing and gloves were evaluated between October and November 2018. The characteristics of the tested pieces are shown in Table 1.

**Table 1:** Main characteristics of the 16 fabrics tested.

Product	Color	Fabric	Brand
T-shirt	Red	Cotton (dense)	GAP
T-shirt	White	Cotton	Hering
T-shirt	Grey	Cotton	Hering
Dry-fit sports t-shirt	Black	Polyamide	Nike
Striped shirt	White and blue	Cotton	Tommy Hilfiger
Polo shirt	Burgundy	Cotton	Ellus
Long-sleeved shirt	Grey	Cotton	Bluesteel
Sweatshirt	Grey	Cotton	GAP
Long dress	Dark blue	Cotton	Hering
Floral dress	Floral print	Polyester	Forever 21
Dark shorts	Blue	Nylon	Lacoste
Short	Light checkerboard	Cotton	Tommy Hilfiger
Legging pants	Black	Polyester (dense)	Domyos
Jeans	Indigo Blue	Denim	TNG
Long-sleeve shirt^a^	Blue	Polyamide	UVLine
Gloves^a^	Beige	Polyamide	UVLine

a Product sold with UV protection in the mesh.

The clothes were submitted to artificial sources of UVB (230 µW/cm^2^; FS72T12/UVB/HO), UVA (1270 µW/cm^2^; Phillips-TL 100 W/10R), and LED light (110 mW/cm^2^ in the blue-violet range; GBRLUX 200 W). Transmittance was assessed by the following devices: UVB Digital Ultraviolet Radiometer (ZooMed – San Luis Obispo, CA, United States), Digital Ultraviolet Radiometer 4.2 UVA (Solarmeter – Glenside, PA, United States) and RD-7 Radiometer (Ecel – Ribeirão Preto, SP, Brazil) after three measurements of each garment.

Table 2 shows the transmittances and UPF for the tested clothing. As for UVB, thin cotton fabrics had the worst performance (UPF < 50). UVA transmittance was also greater in thinner fabric; seven (44%) items presented UPF < 15. Total visible light and blue light were effectively blocked by colored fabrics. The shirt and gloves that claimed to offer UV protection provided adequate blocking for all tested radiation.

**Table 2:** Percentage of UV and visible light transmittance of the different fabrics tested.

Product	UVB (%)	UPF-UVB	UVA (%)	UPF-UVA	tVL (%)	BVL (%)
Red GAP T-Shirt	0.3%	50+	2.5%	41	1.4%	0.0%
Hering white t-shirt	3.8%	27	10.8%	9	42.3%	27.4%
Gray Hering T-Shirt	2.3%	43	6.2%	16	7.5%	0.0%
Dry-fit sports t-shirt	2.5%	41	16.1%	6	3.9%	0.0%
Striped cotton shirt	6.2%	16	17.3%	6	35.4%	29.9%
Burgundy polo shirt	0.5%	50+	2.8%	35	1.0%	0.0%
Gray cotton long sleeve blouse	3.9%	26	17.0%	6	6.8%	0.0%
Gray sweatshirt	0.0%	50+	0.2%	50+	2.9%	0.0%
Dark blue long dress	1.8%	50+	4.3%	24	2.3%	0.0%
Floral dress	4.6%	22	40.8%	2	19.0%	49.1%
Dark nylon shorts	1.4%	50+	12.6%	8	2.2%	0.0%
Light-colored checkered cotton shorts	3.7%	27	10.6%	9	21.5%	31.4%
Legging pants	0.1%	50+	0.6%	50+	0.1%	0.0%
Jeans	0.1%	50+	0.1%	50+	0.1%	0.0%
Long-sleeve shirt^a^	0.3%	50+	0.5%	50+	0.5%	0.0%
Glove	0.2%	50+	1.4%	50+	2.4%	0.0%

UVB, ultraviolet B; UVA, ultraviolet A; UPF, ultraviolet protection factor; LVt, total visible light (400-780nm); BVL, blue-violet light (400-500nm).

a Product sold with UV protection on the mesh.

This study corroborates the findings in the literature that light clothes and thin knits have higher solar radiation transmittance, which should serve as an alert in the recommendation of photoprotection during the practice of outdoor sports or even for guidance on daily sun protection, especially in sunny regions. A fabric with UPF < 15 is considered insufficient for usual protection and, in situations of great exposure (*e.g*., beach, parks, sun exposure during work), clothing with UPF > 50 is recommended. There should be a preference for clothing with dark colors, denser weave (or treated with UV protection), and of adequate size, since stretching of the fibers and proximity to the skin can reduce the protection.^5^

In outdoor activities, children commonly use thin T-shirts, cotton dresses, and shorts, which can distort the perception of protection of covered areas. Moreover, the rate of sun exposure in childhood is an important risk factor for the onset of melanoma in adulthood, being the target of campaigns in several countries.^2^ Likewise, in addition to the appropriate sunscreen, outdoor sports (*e.g.*, running, tennis, soccer, cycling) must be performed with low transmittance clothing, as transmittance can be intensified by the moisture of sweat.

The transmittance of clothing is not parallel to the biological effect of radiation; however, it is a way of comparing different fabrics. Despite the wide availability of coverage and the prompt and stable photoprotection promoted by clothing, a significant portion of the skin remains exposed during the day (*e.g*., face, posterior neck, and hands), requiring additional care, such as hats and sunscreen. Moreover, as much of Brazil has a warm climate, it is usual for young people to wear short clothes, minimizing the protection promoted by clothing.

This study has limitations, as it did not take into account all the variety of colors and fabrics that the industry offers, and did not compare the state of use and moisture of the tested garments.

In conclusion, different clothing fabrics used in Brazil promote variable photoprotection patterns, and this information should be relayed to patients, especially children and those in risk groups.

## Financial support

None declared.

## Authors’ contributions

Melissa Mari Yoshida: Approval of the final version of the manuscript; elaboration and writing of the manuscript; obtaining, analyzing, and interpreting the data; effective participation in research orientation; intellectual participation in propaedeutic and/or therapeutic conduct of studied cases; critical review of the literature; critical review of the manuscript.

Ana Cláudia Cavalcante Esposito: Approval of the final version of the manuscript; elaboration and writing of the manuscript; effective participation in research orientation; critical review of the literature; critical review of the manuscript.

Hélio Amante Miot: Statistical analysis; approval of the final version of the manuscript; conception and planning of the study; elaboration and writing of the manuscript; obtaining, analyzing, and interpreting the data; effective participation in research orientation; intellectual participation in propaedeutic and/or therapeutic conduct of studied cases; critical review of the literature; critical review of the manuscript.

## Conflicts of interest

None declared.
